# Biological therapies in paediatric Behçet’s disease: results of an international collaborative study by the PRES Vasculitis Working Party

**DOI:** 10.1093/rheumatology/keaf404

**Published:** 2025-08-01

**Authors:** Özlem Akgün, Fatma Gül Demirkan, Isabelle Koné-Paut, Taner Coşkuner, Veysel Çam, Merve Cansu Polat, Esra Esen, Emilio Amleto Conti, Nihal Şahin, Özge Baba, Gülşah Kılbaş, Nesibe Gökçe Kocamaz, Sema Nur Taşkın, Donato Rigante, Marija Jelusic, Annacarin Horne, Kübra Öztürk, Semanur Özdel, Oya Köker, Selçuk Yüksel, Mukaddes Kalyoncu, Hafize Emine Sönmez, Giovanni Filocamo, Ayşenur Paç Kısaarslan, Maria Vincenza Mastrolia, Banu Çelikel Acar, Dallel Benazzouz, Ezgi Deniz Batu, Betül Sözeri, David Saadoun, Seza Özen, Nuray Aktay Ayaz

**Affiliations:** Department of Pediatric Rheumatology, School of Medicine, Istanbul University, Istanbul, Turkey; Department of Pediatric Rheumatology, School of Medicine, Istanbul University, Istanbul, Turkey; Department of Paediatric Rheumatology and CEREMAIA, Bicêtre University Paris Saclay Hospital, APHP, Le Kremlin Bicetre, France; Department of Pediatric Rheumatology, Ümraniye Research and Training Hospital, University of Health Sciences, Istanbul, Turkey; Division of Pediatric Rheumatology, Department of Pediatrics, Faculty of Medicine, Hacettepe University, Ankara, Turkey; Division of Pediatric Rheumatology, Department of Pediatrics, Ankara City Hospital, University of Health Sciences, Bilkent, Ankara, Turkey; Department of Pediatric Rheumatology, Erciyes University, Kayseri, Turkey; Pediatric Immuno-Rheumatology Unit, Fondazione IRCSS Ca’ Granda Ospedale Maggiore Policlinico, Milan, Italy; Department of Pediatric Rheumatology, Kocaeli University, Izmit, Turkey; Pediatric Rheumatology, Faculty of Medicine, Karadeniz Technical University, Trabzon, Turkey; Department of Pediatric Rheumatology, Pamukkale University Faculty of Medicine, Denizli, Turkey; Department of Pediatric Rheumatology, Ankara Etlik City Hospital, Ankara, Turkey; Department of Pediatric Rheumatology, Eskişehir City Hospital, Eskisehir, Turkey; Department of Life Sciences and Public Health, Fondazione Policlinico Universitario A. Gemelli IRCCS, Rome, Italy; Department of Life Sciences and Public Health, Università Cattolica Sacro Cuore, Rome, Italy; Department of Pediatrics, University of Zagreb School of Medicine, University Hospital Centre Zagreb, Zagreb, Croatia; Department of Women’s and Children’s Health, Karolinska Institutet Cancerforskning KI, Stockholm, Sweden; Department of Pediatric Rheumatology, Göztepe Research and Training Hospital, Istanbul Medeniyet University, Istanbul, Turkey; Department of Pediatric Rheumatology, Ankara Etlik City Hospital, Ankara, Turkey; Division of Pediatric Rheumatology, Department of Pediatrics, Marmara University School of Medicine, Istanbul, Turkey; Department of Pediatric Rheumatology, Pamukkale University Faculty of Medicine, Denizli, Turkey; Pediatric Rheumatology, Faculty of Medicine, Karadeniz Technical University, Trabzon, Turkey; Department of Pediatric Rheumatology, Kocaeli University, Izmit, Turkey; Pediatric Immuno-Rheumatology Unit, Fondazione IRCSS Ca’ Granda Ospedale Maggiore Policlinico, Milan, Italy; Department of Pediatric Rheumatology, Erciyes University, Kayseri, Turkey; Rheumatology Unit, ERN ReCONNET Center, Meyer Children’s Hospital IRCCS, Firenze, Italy; Division of Pediatric Rheumatology, Department of Pediatrics, Ankara City Hospital, University of Health Sciences, Bilkent, Ankara, Turkey; Department of Paediatric Rheumatology and CEREMAIA, Bicêtre University Paris Saclay Hospital, APHP, Le Kremlin Bicetre, France; Division of Pediatric Rheumatology, Department of Pediatrics, Faculty of Medicine, Hacettepe University, Ankara, Turkey; Department of Pediatric Rheumatology, Ümraniye Research and Training Hospital, University of Health Sciences, Istanbul, Turkey; From Sorbonne Universités Assistance Publique-Hôpitaux de Paris (AP-HP), Groupe Hospitalier Pitié- Salpêtrière, Département de Médecine Interne et Immunologie Clinique, Centre National de Références Maladies Autoimmunes Systémiques Rares, Centre National de Références Maladies Autoinflammatoires et Amylose Inflammatoire, Clinical Investigation Center in Biotherapy, and INSERM 959, Groupe Hospitalier Pitié-Salpêtrière, AP-HP, Paris, France; Division of Pediatric Rheumatology, Department of Pediatrics, Faculty of Medicine, Hacettepe University, Ankara, Turkey; Department of Pediatric Rheumatology, School of Medicine, Istanbul University, Istanbul, Turkey

**Keywords:** Behçet’s disease, biologic therapy, vasculitis, paediatric/juvenile rheumatology, autoinflammatory conditions

## Abstract

**Objective:**

This study aims to examine the indications for paediatric rheumatologists to use biologic therapies in childhood Behçet’s disease (BD), as well as the efficacy, safety and remission rates of such treatments. We also compare demographic and clinical characteristics of Turkish and European cohorts.

**Methods:**

In this multicentric retrospective study, the data of 109 paediatric BD (pedBD) patients diagnosed before 18 years of age, obtained from Pediatric Rheumatology European Society (PRES) vasculitis study group, which involves 19 centres across six countries were analysed.

**Results:**

Of the patients, 64 were from Turkey and 45 from Europe. The primary indications for initiating biologics were ocular (40.4%), mucocutaneous (22.9%) and neurologic (17.4%) involvement. TNF-α inhibitors were used in 90.8% of cases, with adalimumab (ADA, 59 patients) and infliximab (IFX, 38 patients) being the most commonly prescribed agents. Skin involvement was more common in Turkish cohort compared with European cohort (*P* < 0.01), while other organ/system involvement was similar. The interval between symptom onset and diagnosis was longer in the European group. Though not significant, biological therapies were initiated earlier and had longer duration in the European cohort. Remission rates were similar in patients receiving ADA and IFX; however, patients receiving ADA experienced a faster decline in disease activity scores, but the difference was not significant (*P* = 0.2). Only one serious infection was reported, and no malignancy/autoimmune disease were observed.

**Conclusion:**

Biologic therapies exhibit robust efficacy and an acceptable safety profile in pedBD. The study emphasizes ocular, mucocutaneous and neurological involvement as principal indications for initiating biologics and offers regional insights into therapeutic strategies.

## Graphical Abstract

**Figure:**
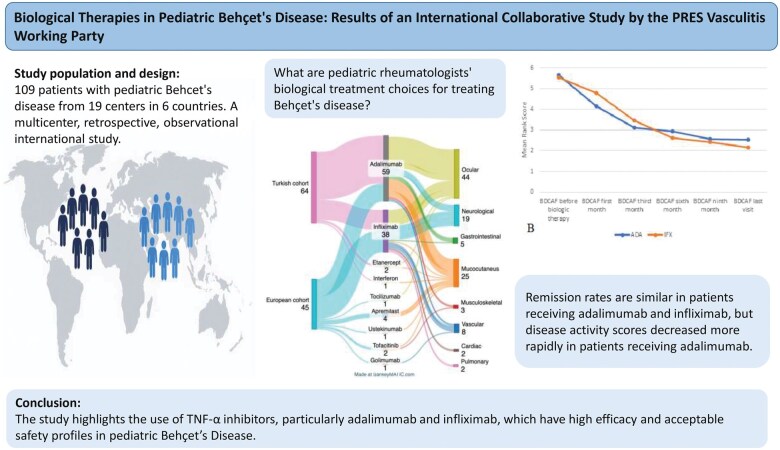

Rheumatology key messages

- TNF-α inhibitors, especially adalimumab, are the most widely used biological therapies in paediatric Behçet’s disease.
- Ocular, mucocutaneous and neurological involvement are the three most common reasons for initiating biologic therapy in paediatric Behçet’s disease patients.
- Biologic therapies show generally favourable efficacy and safety profiles in the treatment of paediatric Behçet’s disease.

## Introduction

Behçet’s disease (BD), first identified by Turkish dermatologist Hulusi Behçet, is a variable vessels’ vasculitis affecting arteries and veins of all sizes, commonly involving the skin, mucosa and occasionally the ocular, vascular, musculoskeletal, neurologic and gastrointestinal (GI) systems. Paediatric cases represent 4–26% of all BD cases, with similar incidence between genders but different clinical presentations [1, 2].

Originally endemic along the historic Silk Road, BD cases have been on the rise worldwide due to migration and better awareness. Diagnosis in children can be challenging due to variable symptoms and delayed phenotype development. While adult criteria like The International Study Group (ISG) and the International Criteria for Behçet’s Disease (ICBD) are widely used, the paediatric BD (pedBD) criteria introduced in 2015, though specific, have low sensitivity. Expert opinion remains still essential for diagnosis [3–5].

BD is defined as an MHC-1opathy, which involves both innate and adaptive immunity [6]. HLA-B51 plays a pivotal role in this process. Interaction with ERAP1 disrupts regulatory T cell homeostasis, leading to an increase in Th1 and Th17 effector cells. Furthermore, pro-inflammatory macrophages (M1) contribute to the polarization of Th1. Elevated cytokines, including IL-1, IL-6 and TNF-α, underline disease activity, making biologics targeting these cytokines promising treatments [7, 8]. Anti-TNF-α therapy is highly effective in refractory cases with severe organ involvement, while IL-1 and IL-6 inhibitors show potential, but data on their use in children are limited [9].

Treatment is tailored to the severity of the disease and the organs involved, based on adult recommendations such as the The European Alliance of Associations for Rheumatology (EULAR) 2018 guidelines. Goals include controlling inflammation, relieving symptoms and preventing complications through individualized approaches [10].

This overview aims to explore biologic therapy in pedBD, evaluating efficacy, side effects and remission rates, and comparing demographic and clinical findings between Turkish and European cohorts.

## Methods

### Study population

This multicentre, retrospective, observational international study, conducted from 1 January 2022 to 31 December 2023, involved 19 centres across six countries within the PRES vasculitis working group. It included patients diagnosed with pedBD [3] and/or ICBD [11] classification criteria before age 18 who received at least 3 months of biologic therapy.

Patients diagnosed after age 18, those not meeting BD criteria or receiving biologics for comorbid conditions were excluded. Of the 116 patients enrolled, seven were excluded due to incomplete criteria, treatment duration under three months or missing data, leaving 109 for final analysis. Patients were grouped into Turkish and European cohorts for comparison of demographic and clinical features, laboratory data and biologic therapy choices. The efficacy, side effects and treatment modifications of biologic therapies were also analysed.

The study was approved by the Istanbul Faculty of Medicine Ethics Committee and conducted in accordance with the Declaration of Helsinki (1653276). Written consent was obtained and is archived according to our institutional policy.

### Definition of organ involvement

Mucocutaneous and skin involvement included recurrent oral and genital ulcers, as well as erythema nodosum, pseudofolliculitis, papulopustular or acneiform lesions. Ocular findings comprised anterior, posterior or panuveitis and retinal vasculitis. Vascular involvement included venous and arterial thrombosis and aneurysms. Dural sinus thrombosis, classified as neurologic or vascular, was considered single-site involvement. Musculoskeletal symptoms encompassed peripheral arthritis, arthralgia or myalgia. Neurologic involvement required MRI findings consistent with BD-related symptoms, while gastrointestinal involvement was confirmed by endoscopy.

### Other definitions

In our study, the term biological therapy includes biological agents and small molecule therapies.

The Behçet’s Disease Current Activity Form (BDCAF) evaluates clinical features observed within the four weeks before the visit, focusing on organ involvement such as headache, ulcers, skin lesions, joint symptoms, gastrointestinal issues, ocular and nervous system involvement and vascular issues [12]. Only new symptoms attributed to BD were recorded.

Remission was defined as a normal acute phase response, the absence of constitutional symptoms, steroid discontinuation or reduction to <10 mg/day, improvement in affected systems and a BDCAF score <1 for at least 6 months. Partial remission involved partial clinical, laboratory and imaging improvement, a 50% steroid dose reduction and a lower BDCAF index. Refractory/severe disease or exacerbation was marked by new or worsening system involvement, increased acute phase reactants or a higher BDCAF index [13, 14].

Drug hypersensitivity reactions are immune-mediated adverse reactions that can be classified as early (e.g. anaphylaxis, urticaria) or delayed (e.g. maculopapular rash, vasculitis, Stevens–Johnson syndrome).

Infections related to drug side effects were categorized as mild (e.g. common cold, mild influenza), moderate (requiring treatment but not hospitalization, such as urinary tract infections or mild pneumonia) and severe (requiring hospitalization or urgent care, such as sepsis or severe pneumonia).

Patients were categorized by ethnicity: White (European), Black (African), Middle Eastern, Turkish or Asian, with the Turkish cohort identified separately due to genetic diversity. We presented this group separately from other ethnic groups because of the significantly higher prevalence of BD in the Turkish population and its different clinical and genetic characteristics (e.g. HLA-B51 association).

### Statistical analysis

Statistical analysis was performed using Statistical Package for Social Sciences IBM (SPSS-IBM), version 22 (SPSS Inc., Chicago, Illinois, USA). Descriptive statistics, including minimum, maximum, median, mean and standard deviation, were calculated based on the distribution of variables. Variables were evaluated for normal distribution using visual and analytical methods. Histogram, Kolmogorov–Smirnov test or Shapiro–Wilk test, and *Q*–*Q* plots were used to assess the normal distribution of continuous variables.

Categorical variables were analysed using Fisher’s exact test or the *χ*^2^ test, while continuous variables were examined with Student’s *t*-test or the Mann–Whitney *U*-test, as appropriate. Two-way analysis of variance by ranks (Friedman’s test) was used when more than two dependent variables were present, and the data were not normally distributed.

## Results

### Demographic characteristics in cohort

A total of 109 pedBD patients were included in the study, 45 in the European cohort and 64 in the Turkish cohort. Males comprised 64.2% of the overall cohort. The median age at diagnosis in the entire cohort was 13.1 years (IQR25p–75p: 10–15.4) and the median follow-up was 32.5 months (IQR25p–75p: 19.2–58.9).

A significant difference was noted in diagnostic delay, with a longer median delay in the European cohort (15.5 months) compared with the Turkish cohort (7.5 months) (*P* = 0.03). Although biologics were initiated earlier and used for a longer duration in the European cohort, the differences were not statistically significant (*P* = 0.06, *P* = 0.23). Comorbidities were more prevalent in the European cohort (*P* = 0.03). Details of clinical and demographic data for the European and Turkish cohorts are presented in Table 1.

**Table 1.** Demographic characteristics of paediatric Behçet’s disease patients in the European and Turkish cohorts

	Turkish cohort (*n* = 64)	European cohort (*n* = 45)	*P-*value
Gender (female/male)	19/45	20/25	0.11*
Current age (years), mean ± S.D.	16.3 ± 4.2	17.3 ± 4.4	0.24**
Age at diagnosis (years), median (IQR25p–75p)	13.3 (10.2–15.3)	13.1 (9.3–15.9)	0.76***
Follow-up duration (months), median (IQR25p–75p)	31.3 (18.2–52.7)	33 (21.4–81.7)	0.26***
Delay in diagnosis (months), median (IQR25p–75p)	7.5 (2.1–22.5)	15.5 (2.2–47.3)	**0.03** ***
Follow-up duration under biological therapy (months), median (IQR25p–75p)	18 (9–26)	25 (8.2–46.5)	0.23***
Follow-up duration until receiving biological therapy (months), median (IQR25p–75p)	8 (3–19.2)	4.5 (1–13.2)	0.06***
History of BD in the family, *n* (%)	14 (21.9)	10 (22.2)	0.69*
HLA-B51 positivity, *n*/*N* (%)	38/60 (63.3)	18/35 (51.4)	0.17*
Pathergy test positivity, *n*/*N* (%)	17/58 (29.3)	0/6 0	0.18*
Comorbid disease, *n*/*N* (%)	9/64 (14.1)	14/45 (31.5)	**0.03** *
Race/ethnicity			
White (European), *n*/*N* (%)	0 0	34/44 (77.2)	
Black (African), *n*/*N* (%)	0 0	5/44 (11.3)	
Middle East, *n*/*N* (%)	1/64 (1.6)	1/44 (2.2)	
Turkish, *n*/*N* (%)	63/64 (98.4)	1/44 (2.2)	
Asian, *n*/*N* (%)	0 0	3/44 (6.8)	

Bold text indicates statistically significant *P*-values (*P* < 0.05).

* Comparisons were performed using the *χ*^2^ test.

** Comparisons were performed using the Student’s *t*-test.

*** Comparisons were performed using the Mann–Whitney *U*-test.

SD, standard deviation; IQR, interquartal range; HLA, human leukocyt antigen.

In the European cohort, comorbidities included type 1 diabetes mellitus, recurrent streptococcal pyogenes infection, epilepsy, depression, MEFV heterozygous mutation, Pierre Robin Syndrome, epilepsy, asthma, enthesitis-related arthritis (ERA) and pseudohyperparathyroidism, and in the Turkish cohort, FMF, acute rheumatic fever, chronic nonbacterial osteomyelitis, ERA, acute disseminated encephalomyelitis and foot drop.

### The distribution of clinical findings at diagnosis

Skin manifestations were significantly higher in the Turkish cohort (68.8% vs 44.4%, *P* = 0.01). Oral aphthae and ocular involvement were more common in the Turkish cohort, but not statistically significant (*P* = 0.12, *P* = 0.07). Other organ/system involvement including pulmonary, cardiac, musculoskeletal (arthritis, arthralgia) and urogenital (epididymo-orchitis) was observed in 45.3% of the Turkish cohort and 31.8% of the European cohort (*P* = 0.22). A more detailed comparison of clinical characteristics is shown in Table 2.

**Table 2.** Comparison of the involved sites and side effects according to the classification criteria in paediatric Behçet’s disease patients in Turkish and European cohorts

	Turkish cohort (*n* = 64)	European cohort (*n* = 45)	*P*-value
Oral aphthae, *n* (%)	62 (96.8)	40 (88.9)	0.12*
Genital ulcer, *n* (%)	31 (48.4)	20 (44.4)	0.82**
Ocular involvement, *n* (%)	38 (59.4)	18 (40)	0.07**
Anterior uveitis, *n*	8	6	
Posterior uveitis, *n*	6	0	
Panuveitis, *n*	18	9	
Retinal vasculitis, *n*	4	3	
Intermediate uveitis, *n*	4	1	
Neurological involvement, *n* (%)	17 (26.6)	15 (33.3)	0.58**
Parenchymal type, *n*	1	8	
Non-parenchymal type, *n*	15	5	
Mixed type, *n*	1	2	
Vascular involvement, *n* (%)	21 (32.8)	12 (26.6)	0.63**
Venous thrombosis, *n*	19	9	
Arterial thrombosis, *n*	1	2	
Arterial aneurysm, *n*	1	0	
Skin involvement *n* (%)	44 (68.8)	20 (44.4)	**0.01** **
Erythema nodosum, *n*	17	5	
Pseudofolliculitis and/or papulopustular lesions, *n*	21	14	
Acneiform nodules, *n*	11	1	
Side effect			
Number of side effects, *n*	8	**7**	0.85***
Infections, *n*	6	2	0.46*
Activation of tuberculosis, *n*	2	0	0.51*
Mild infections, *n*	2	2	1*
Moderate infections, *n*	2	1	1*
Severe infections, *n*	1	0	1*
Injection site reactions, *n*	1	2	0.56*
Hypersensitivity reactions, *n*	2	3	0.39*
Demyelinating disease, *n*	1	0	1*

Bold text indicates statistically significant *P*-values (*P* < 0.05).

* Comparisons were performed using the Fisher’s exact test.

** Comparisons were performed using the continuity correction.

*** Comparisons were performed using the *χ*^2^ test.

### Initial evaluation of first-choice biological therapies

In the overall cohort, ocular involvement (44 cases, 40.4%) was the most common indication for biologics, followed by mucocutaneous (25 cases, 22.9%) and neurologic involvement (19 cases, 17.4%). TNF-α inhibitors were the most frequently used biologics (99 cases, 90.8%), with adalimumab (ADA) (59 cases) as the first choice, followed by infliximab (IFX) (38 cases).

**Figure 1.**
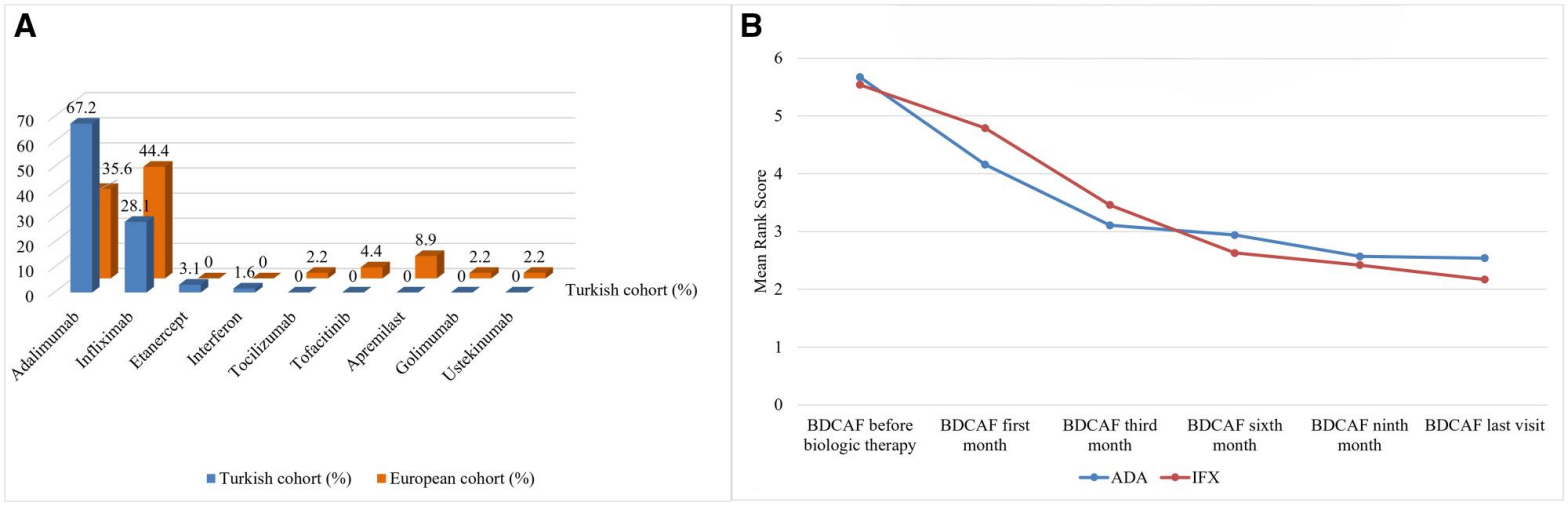
First-choice biologic therapies in Turkish/European cohorts and ADA/INF effect on BDCAF scores in entire cohort. (**A**) First-choice treatments in Turkish and European paediatric Behçet’s disease cohorts. (**B**) Effect of ADA and INF treatments on BDCAF score. BDCAF, Behçet’s Disease Current Activity Form; ADA, adalimumab; IFX, Infliximab

**Figure 2.**
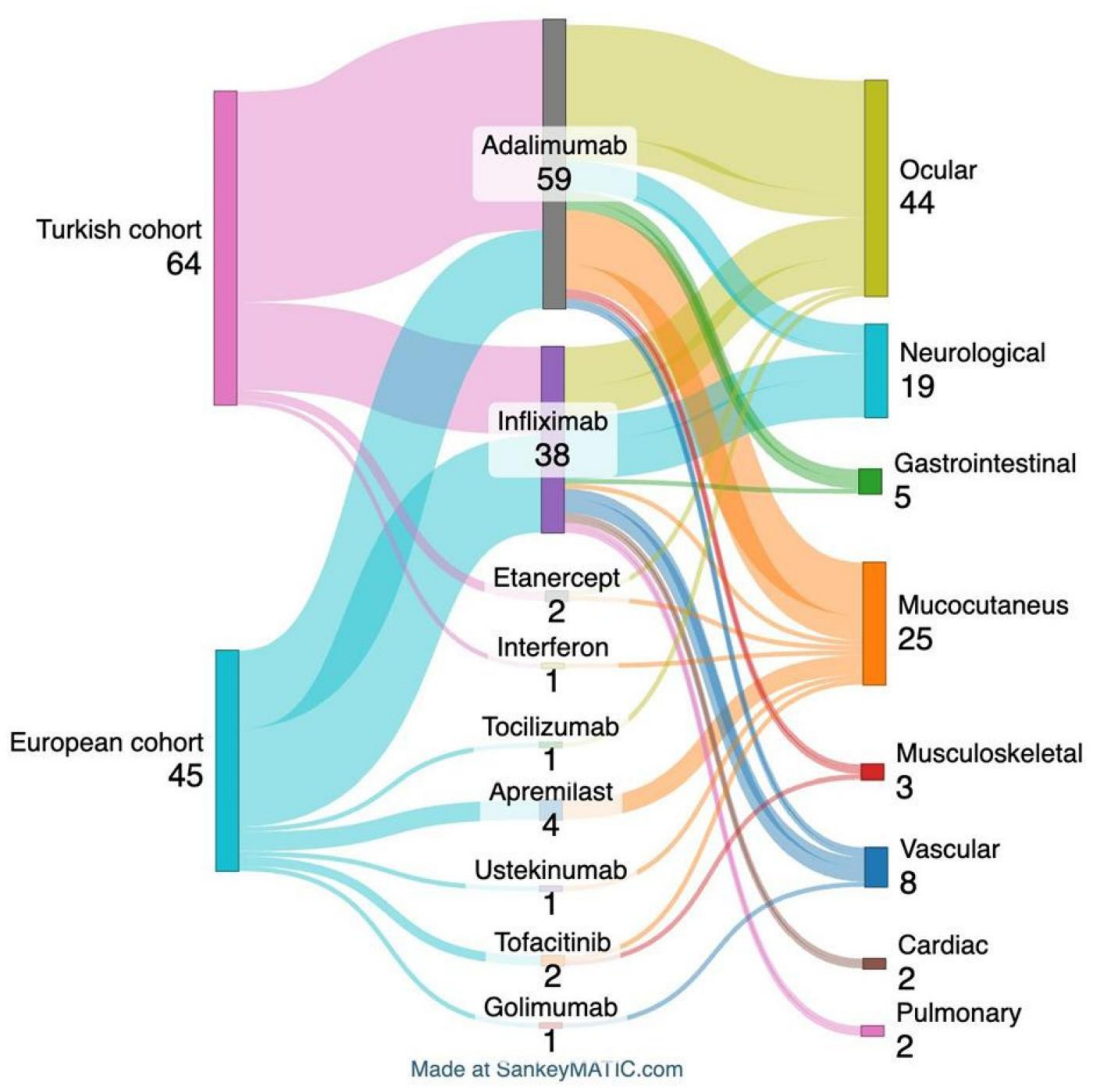
Sankey diagram summarizing the drugs choices by the groups and the reasons for their choices

For ADA, ocular involvement (28 cases, 47.5%) was the leading indication, followed by mucocutaneous (16 cases, 27.1%) and neurologic (6 cases, 10.2%) involvement. Similarly, IFX was primarily used for ocular (14 cases, 36.8%), neurologic (13 cases, 34.2%) and vascular (five cases, 13.2%) involvement, with specific choice for cardiac and pulmonary manifestations. Detailed data on biologic drug usage and indications in each cohort are shown in Fig. 1A and Table 3. The drug choices for patients in the Turkish and European cohorts, as well as the indications for which they were chosen, are summarized in Fig. 2.

**Table 3.** First-choice biologic therapies according to organs/systems in Turkish and European cohorts

	Turkish cohort (*n* = 64)	European cohort (*n* = 45)	*P-*value
Ocular involvement	32 (50)	12 (26.7)	0.11*
Adalimumab, *n* (%)	23 (71.9)	5 (41.7)	
Infliximab, *n* (%)	8 (25)	6 (50)	
Etanercept, *n* (%)	1 (3.1)	0 0	
Tocilizumab, *n* (%)	0 0	1 (8.3)	
Neurological involvement	10 (15.6)	9 (20)	0.06*
Adalimumab, *n* (%)	5 (50)	1 (12.5)	
Infliximab, *n* (%)	5 (50)	8 (88.9)	
Vascular involvement	3 (4.7)	5 (11.1)	0.68**
Adalimumab, *n* (%)	1 (33.3)	1 (20)	
Infliximab, *n* (%)	2 (66.7)	3 (60)	
Golimumab, *n* (%)	0 0	1 (20)	
Mucocutaneous involvement	13 (20.3)	12 (26.7)	0.08*
Adalimumab, *n* (%)	11 (84.6)	5 (41.7)	
Infliximab, *n* (%)	0 0	1 (8.3)	
Apremilast, *n* (%)	0 0	4 (33.3)	
Tofacitinib, *n* (%)	0 0	1 (8.3)	
Etanercept, *n* (%)	1 (7.7)	0 0	
Interferon, *n* (%)	1 (7.7)	0 0	
Ustekinumab, *n* (%)	0 0	1 (8.3)	
Gastrointestinal involvement	3 (4.7)	2 (4.4)	0.36**
Adalimumab, *n* (%)	2 (66.7)	2 (100)	
Infliximab, *n* (%)	1 (33.3)	0 0	
Musculoskeletal involvement	1 (1.6)	2 (4.4)	0.51**
Adalimumab, *n* (%)	1 (100)	1 (50)	
Tofacitinib, *n* (%)	0 0	1 (50)	
Cardiac involvement	1 (1.6)	1 (2.2)	–
Infliximab, *n* (%)	1 (100)	1 (100)	
Pulmonary involvement	1 (1.6)	1 (2.2)	–
Infliximab, *n* (%)	1 (100)	1 (100)	

* Comparisons were performed using the *χ*^2^ test.

** Comparisons were performed using the Fisher’s exact test.

In the European cohort, IFX was mostly used (44.4%), followed by ADA (35.6%) and apremilast (8.9%). In contrast, in the Turkish cohort, ADA was chosen (67.2%), followed by IFX (28.1%) and ETN (3.1%).

### First-choice biological therapies for organ/system involvement

In the European cohort, IFX (50%) was mostly used for ocular involvement, while ADA (71.9%) was in the Turkish cohort. One Turkish patient started on ETN for ocular involvement later switched to ADA. In the European cohort, tocilizumab (TCZ) was used for ocular involvement in one patient.

Among patients with neurological involvement, IFX was the predominant first-line biologic therapy used in the European cohort, administered to eight out of nine patients (88.9%), whereas ADA was used in only one case. In contrast, the Turkish cohort showed an equal distribution, with five patients treated with ADA and five with IFX.

For mucocutaneous involvement, five out of 12 European patients received ADA, and four received apremilast, while 11 out of 13 Turkish patients (84.6%) were treated with ADA.

In both cohorts, IFX was the biologic of choice for cardiac and pulmonary involvement. Detailed organ involvement and treatment choices are provided in Table 3.

### Clinical outcomes

In the European cohort, two patients (4.4%) were in drug-free remission, 22 patients (48.9%) were in remission with drugs, 12 patients (26.7%) were in partial remission and six patients (13.3%) had refractory disease. Follow-up clinical outcome data were missing for three patients (6.7%). In the Turkish cohort, one patient (1.6%) was in drug-free remission, 49 patients (76.6%) were in remission with drugs, eight patients (12.5%) were in partial remission and three patients (4.7%) had refractory disease. Follow-up clinical outcome data were missing for three patients (4.7%). The Turkish cohort had a higher remission rate with drug treatment compared with the European cohort (*P* = 0.02). When comparing the two mostly used biologics, ADA and IFX, there was no significant difference in BDCAF, CRP or ESR levels between the groups. At the last follow-up, these values were significantly lower than the initial values in both the groups (*P* = 0.001). Remission rates were similar in patients receiving ADA and IFX. Disease activity decreased more rapidly in patients receiving ADA, but the difference was not statistically significant (Fig. 1B).

### Switched biological therapies

The need for biological therapy switching was observed in 16 patients in the European cohort and 11 patients in the Turkish cohort. The frequency of switching and the variety of biologic therapies switched were higher in the European cohort compared with the Turkish cohort.

When the switched treatments were analysed, 6 patients receiving IFX for ocular involvement were switched to ADA due to uveitis attack. One patient receiving IFX for neurologic involvement was switched to ADA due to problems in drug supply and the other patient was switched to ADA due to refractory disease. The patient receiving IFX for mucocutaneous involvement was switched to ADA due to relapses.

One of the patients who was started on ADA for ocular involvement was switched to IFX due to refractory disease and the other patient was switched to IFX for mucocutaneous involvement and then to INF for vascular involvement. Among the five patients who were started on ADA for mucocutaneous involvement, two were switched to ETN due to hypersensitivity reaction, two to IFX due to relapse of the disease and one to GOL because complete remission was not achieved. Among the two patients who were started on ADA due to musculoskeletal involvement, one was switched to IFX due to refractory disease, and the other was switched to GOL due to ocular involvement.

The biological therapies switched, the cohort to which the switch belonged and the reasons for switching are detailed in Supplementary Table S1.

### Side effects related to biological therapies

The frequency of side effect due to biologics was similar between the groups. In the European cohort, no cases of tuberculosis reactivation or serious infections were reported. However, in the Turkish cohort, two patients receiving ADA experienced tuberculosis reactivation, and one developed a serious infection. In the European cohort, two patients on IFX for neurological and pulmonary issues developed hypersensitivity reactions and were switched to ADA and TCZ, respectively. In the Turkish cohort, two patients on ADA for mucocutaneous involvement were switched to ETN due to hypersensitivity reactions. One patient on TCZ for ocular involvement had hypersensitivity but continued with the treatment. Three patients had injection site reactions: two in the European cohort on IFX and one in the Turkish cohort on ADA; no changes in treatments were made. No cases of malignancy or autoimmune disease were reported in the cohort. Detailed side effect information is provided in Table 2.

## Discussion

This study provides real-life data on the use of biologic therapies in a large group of pedBD across several European countries, focusing on treatment patterns and indications. We also highlighted the differences in clinical and demographic data, as well as clinicians’ choices for biologic therapies, between Turkey—the country with the highest BD prevalence—and other European countries. While previous studies have mainly looked at biologics for specific organ involvement, to our knowledge, this is the first multicentre, international study to comprehensively assess biologic therapy in pedBD patients. In this study, the three most common reasons for initiating biologic therapy in pedBD were ocular, mucocutaneous and neurological involvement. Biologic therapies have generally demonstrated favourable efficacy and safety profiles in pedBD. TNF-α inhibitors were the most commonly used biologic therapies, with ADA being the preferred choice, followed by IFX. Remission rates were similar in ADA-treated patients and IFX-treated patients, although patients receiving ADA had a faster decline in BDCAF scores, but this difference was not statistically significant.

Based on pedBD cohorts in the literature, the mean age of onset ranges from 5 to 13 years, with no significant gender difference [15–17]. In our study, the age at diagnosis aligns with the literature, but the male sex was more prevalent, particularly in the Turkish cohort. Since males typically experience a severe course of BD, this is not surprising, as biologic-treated patients usually have a severe disease. Moreover, male and female patients may show differences in presentation and symptom severity, reflecting genetic and environmental factors [18, 19]. For instance, ocular and vascular symptoms are common in boys, while mucocutaneous involvement, such as oral and genital ulcers, is more frequent in girls [1].

The time from symptom onset to diagnosis was longer in the European cohort than in the Turkish cohort. This may be due to the higher prevalence of the disease in Turkey, where clinicians likely have a lower threshold for suspicion, leading to earlier diagnoses. Although not statistically significant, biologics were initiated earlier and used for a longer duration in the European cohort compared with the Turkish cohort. These differences may reflect variations in clinical practice, healthcare systems and access to biologics.

The geographical location has been demonstrated to exert a significant influence on the clinical presentation of numerous rheumatic diseases. Similarly, the clinical manifestations of pedBD have been observed to exhibit considerable regional variability [20, 21]. Our study found a higher frequency of skin involvement in the Turkish cohort compared with the European cohort. Although no statistical difference was observed between the two cohorts regarding other organ involvement, ocular involvement and oral aphthae were reported at higher rates in the Turkish cohort. In previous studies, a higher frequency of ocular involvement in regions where the disease is more prevalent has been documented [22].

In a systematic review evaluating the use of biologic therapies in pedBD, anti-TNF inhibitors emerged as the most commonly used agents. The primary indication for initiating biologic therapy was ocular involvement, which is consistent with our study’s findings. However, unlike our study, the review identified interferons as the second most commonly used biologic therapy, with multisystem active disease being the second most common indication for their use [23].

Ocular involvement is one of the earliest recognized manifestations of BD. The treatment of uveitis and the prevention of recurrence represent a significant challenge, making ocular involvement the primary indication for biological therapies [24, 25]. Most of the studies on ocular involvement focus on anti-TNF agents, especially ADA and IFX. A study of 177 patients with refractory uveitis due to BD (103 on IFX and 74 on ADA) revealed that both biologics were effective, but ADA demonstrated better outcomes, including higher drug retention rates after one year [26]. In our study, the most preferred biologic drug for ocular involvement was ADA, with a rate of 71.9% in the Turkish cohort, and IFX, with a rate of 50% in the European cohort. Choices for biologic drugs may be influenced by several factors, including drug availability, insurance coverage and the tendency of the physicians to select the most widely promoted drug in their country.

The mucocutaneous manifestations of BD, including oral and genital ulcers, erythema nodosum, papulopustular lesions, superficial thrombophlebitis and pathergy reactions, are diverse and may affect disease outcome depending on the type of lesion. Interestingly, mucocutaneous involvement was the second most common reason for initiating biologic therapy in our study. In the European cohort, the biologics most frequently prescribed for mucocutaneous involvement were ADA (47.6%) and apremilast (33.3%). Other biologics used included IFX, tofacitinib and ustekinumab. In the Turkish cohort, ADA (84.6%) was the dominant choice. A study on the pathophysiology of mucocutaneous involvement found differences in cytokine levels and variety depending on the type of lesion [27]. This may explain the diversity in the use of biological therapies in mucocutaneous involvement and the multiplicity of switches to biological therapies in this involvement.

Neurological involvement in BD presents significant clinical challenges, with an aggressive course and high morbidity and mortality. Prompt and aggressive treatment is of the utmost importance in influencing the clinical outcome. Research has demonstrated that TNF-α is a key pro-inflammatory cytokine in neuro-BD, predominantly produced by macrophages, NK and T cells, playing a significant role in neutrophil activation, contributing to chronic vascular inflammation and multiorgan tissue damage [28, 29]. In both cohorts, ADA and IFX were preferred biologics for neurological involvement. In the European cohort, 88.4% of patients received IFX, while in the Turkish cohort, both ADA and IFX were equally preferred. In a study of 53 patients with neuro-BD, 22 of the patients started infliximab treatment and 21 patients (95%) who completed the first year of treatment achieved remission and disease stabilization. A literature review highlights studies on anti-TNF agents (IFX, ADA, ETN) and anti-IL6 (TCZ) for managing neurological involvement in BD [30–33].

Vascular involvement in BD is distinct from other autoimmune inflammatory conditions, characterized by thrombosis with vascular wall inflammation not explained by thrombophilia factors. Neutrophilic vasculitis, typically affecting the vasa vasorum, is commonly seen in the vessels [34]. The cornerstone of managing vascular involvement in BD is effective control of the underlying inflammatory process. In an adult study evaluating the efficacy and safety of infliximab (IFX), 127 BD patients with vascular involvement (80% male) were treated with IFX. Among them, 87% received IFX for remission induction, and most were already receiving immunosuppressive therapy at the time of vascular involvement. The remission rate following IFX treatment was 73% at 6 months and 63% at 12 months [35]. In the present study, analysis of the indications for initiating biological therapy revealed that vascular involvement accounted for treatment initiation in three patients from the Turkish cohort and five patients from the European cohort. In our patients, anti-TNF agents were preferred in cardiac and pulmonary involvement, in line with the literature [36–38]. Furthermore, clinicians preferred to intervene with pulse methylprednisolone and cyclophosphamide rather than change biological therapy in relapses of these patients.

GI involvement of BD is driven by excessive TNF-α production leading to intense mucosal and submucosal inflammation and ulceration. Monoclonal antibodies bind to soluble and membrane-bound TNF-α, neutralizing its pro-inflammatory effects. ETN is a fusion protein that neutralizes only soluble TNF-α but has limited activity against membrane-bound TNF-α and does not induce apoptosis [39, 40]. Both cohorts preferred ADA and IFX for biologic treatment of GI involvement. This is consistent with international treatment recommendations for BD with GI involvement [10].

There is no data to suggest which biologic therapy is more effective in pedBD. Although patients receiving ADA had faster declines in BDCAF scores, the difference was not statistically significant and remission rates were similar between the two treatments. Furthermore, our data suggest that the safety profile of biologic therapy in pedBD is acceptable [41, 42].

The main limitation of this study is its retrospective design, which may give rise to biases due to reliance on medical records and a lack of control over confounding factors. Furthermore, the lack of standardized protocols for diagnosis, treatment and follow-up represents a limitation in terms of the reliability of comparisons, with variations across centres potentially affecting the generalizability of findings. However, this study, accompanied by data from many centres, will shed light on the treatment of pedBD patients with biological therapies in a large cohort.

## Conclusion

BD is a complex and heterogeneous disease with a wide spectrum of manifestations, ranging from mild mucocutaneous involvement to severe, life-threatening vascular or neurological complications. This international study reviews the indications that lead to the indication for biologic therapy in childhood BD. Our data confirm that biologics can be safely used in BD. Prospective comparative studies with large patient cohorts and long-term follow-up are essential to elucidate the role of biologic treatments in pedBD, in regard to different system involvement.

## Supplementary Material

keaf404_Supplementary_Data

## Data Availability

The data that support the findings of this study are available from the corresponding author, [Prof. Dr Nuray Aktay Ayaz], upon reasonable request. Due to ethical restrictions regarding patient confidentiality, data cannot be made publicly available. Interested researchers may contact [nurayaktay@gmail.com] for data access requests, which will be reviewed on a case-by-case basis following appropriate institutional approval.
