# Supplementary material for: Biological therapies in paediatric Behçet’s disease: results of an international collaborative study by the PRES Vasculitis Working Party
Source: Rheumatology (Oxford). 2025 Aug 1;65(3):keaf404. doi: 10.1093/rheumatology/keaf404 (PMC13016814; doi:10.1093/rheumatology/keaf404)
Supplement: keaf404_Supplementary_Data [file keaf404_supplementary_data.docx]

**Supplementary Table S1.** Switched biological agents in both groups and their reasons

| **Group** | **First selected biologic agent** | **Reason** | **Switched biological agent** | **Reason** | **Other switched**  **biological**  **agents** | **Reason** |
| --- | --- | --- | --- | --- | --- | --- |
| **Turkish (n=6)** | IFX | Ocular | ADA | Attack of uveitis |  |  |
| **Turkish (n=1)** | ETN | Ocular | ADA | Attack of uveitis |  |  |
| **Turkish**  **(n=1)** | IFX | Neurological | ADA | Problems with drug supply |  |  |
| **Turkish (n=1)** | ADA | Ocular | IFX | Mucocutaneous | IFN | Thrombophlebitis |
| **Turkish (n=2)** | ADA | Mucocutaneous | ETN | Hypersensitivity reaction |  |  |
| **European**  **(n=1)** | ADA | GIS | Anakinra | Mucocutaneus | CAN | Refractory disease |
| **European (n=2)** | ADA | Mucocutaneous | IFX | Relapse of disease |  |  |
| **European**  **(n=1)** | ADA | Mucocutaneous | GOL | Partial remission |  |  |
| **European**  **(n=1)** | ADA | Musculoskeletal | IFX | Refractory disease |  |  |
| **European (n=1)** | ADA | Ocular | IFX | Refractory disease |  |  |
| **European (n=1)** | Tofacitinib | Musculoskeletal | Guselkumab | Relapse of arthritis |  |  |
| **European (n=1)** | UST | Mucocutaneous | Apremilast | Refractory disease | ADA, GOL,  IFX, TCZ, UST | Refractory disease |
| **European**  **(n=1)** | Apremilast | Mucocutaneous | UST | Mucocutaneous |  |  |
| **European**  **(n=1)** | Apremilast | Mucocutaneous | GKB | Mucocutaneous |  |  |
| **European**  **(n=1)** | GOL | Vascular | ADA | Relapse of disease | Anakinra  Apremilast |  |
| **European**  **(n=2)** | IFX | Neurological | ADA | Refractory disease/hypersensitivity reaction |  |  |
| **European**  **(n=1)** | IFX | Mucocutaneous | ADA | Relapse of disease |  |  |
| **European**  **(n=1)** | IFX | Ocular | Anakinra | Attack of uveitis | TCZ | Partial remission |
| **European**  **(n=1)** | ADA | Musculoskeletal | TCZ | Ocular |  |  |

*ADA, adalimumab; ETN, etanercept; GOL, golimumab; IFX, infliximab, TCZ, tocilizumab; UST, ustekinumab; IFN, interferon; CAN, canakinumab*
